# 2D Strain Analysis in Myocarditis—Can We Be Any Closer to Diagnose the Acute Phase of the Disease?

**DOI:** 10.3390/jcm12082777

**Published:** 2023-04-08

**Authors:** Karolina Supeł, Paulina Wieczorkiewicz, Katarzyna Przybylak, Marzenna Zielińska

**Affiliations:** Department of Interventional Cardiology, Medical University of Lodz, 90-419 Lodz, Poland

**Keywords:** two-dimensional speckle-tracking echocardiography, cardiac magnetic resonance, acute myocarditis

## Abstract

Background: The aim of present study was to assess left ventricular myocardial deformation detected by 2D STE in patients with suspected acute myocarditis (AM) early on admission in whom later cardiac magnetic resonance (CMR) evaluation was performed. Methods: A total of 47 patients with suspected AM based on clinical practice were prospectively enrolled. Coronary angiography was performed on all patients to rule out significant coronary artery disease. CMR confirmed myocardial inflammation, oedema, and regional necrosis meeting the Lake Louise criteria in 25 patients (53%, oedema (+) subgroup). In the remaining patients, only LGE was confirmed in the sub-epicardial or intramuscular localization (22 patients, 47%, oedema (−) subgroup). Early on admission, echocardiography with measurements of global and segmental longitudinal strains (GLS), circumferential strains (GCS) at the endocardial (endocardial GCS) and epicardial (epicardial GCS) layers, transmural GCS, and radial strains (RS) were performed. Results: Mild reduction of GLS, GRS, and transmural GCS values were found in patients with oedema (+) subgroup. The epicardial GCS turned out to be the diagnostic factor for oedema with a cut-off point of 13,0% (AUC 0.747, *p* = 0.0005). Twenty-two patients (all but three) with an acute phase of myocarditis and epicardial GCS −13.0% or less had oedema confirmed by CMR. Conclusions: 2D STE can help to set the diagnosis of AM in patients with acute chest pain with a normal coronary angiogram. The epicardial GCS can serve as a diagnostic factor for oedema in patients with early stage of AM. In patients presenting with signs of AM (oedema in CMR), the epicardial GCS is modified in comparison with a subgroup without oedema; therefore, this parameter could be used to improve the performance of ultrasound.

## 1. Introduction

Acute myocarditis (AM) is an inflammatory disease of the myocardium usually caused by viral infection. The clinical presentation of AM is heterogenous and includes an asymptomatic course, mimicking acute coronary syndrome, severe acute heart failure, ventricular arrhythmia, or sudden death presentation. Therefore, the prognosis varies in the above-mentioned clinical scenarios. AM was reported in up to 12% of cases of sudden cardiac deaths, with arrhythmia as the most common underlying mechanism [1]. A total of 30% of patients after myocarditis developed secondary dilated cardiomyopathy [2]. Little is known about the outcome of patients with a mild course of the disease, especially in those with preserved left ventricular (LV) function. Despite a benign prognosis of such an AM descent, novel echocardiography methods including strain analysis detect subtle LV dysfunction several months after the acute episode of non-severe AM [3].

The gold standard for the diagnosis of myocarditis was endomyocardial biopsy (EMB) in the past and more recently, cardiac magnetic resonance (CMR). However, endomyocardial biopsy has a low yield due to often patchy involvement of the myocardium [4,5,6] and carries a risk of serious complications (tamponade 0.08%, third-degree atrioventricular block 1.47% [7]). On the other hand, CMR cannot be performed in all cases due to its contraindications (e.g., implanted metal elements in the body) or due to unavailability in the acute phase of AM. The diagnosis of myocarditis by CMR depends on meeting at least two of the three Lake Louise criteria I, which consist of tissue characterization findings including oedema, hyperemia/capillary leak, and necrosis [2,3], or two of the two Updated Lake Louise criteria II which consist of myocardial oedema in T2 mapping or T2W images and non-ischemic myocardial injury—abnormal T1, ECV, or LGE [8]. Inflammatory cardiac MRI parameters decreased over time from acute, sub-acute to chronic, or healed disease. In the Myo-Racer study, only T2 mapping out of the conventional Lake Louise criteria and novel mapping techniques was found to be sufficiently diagnostic in chronic myocarditis, with an AUC of 0.77, higher than for the Lake Louise criteria I (0.53) and native T1 (0.53) [8]. Experts recommend that having only one (T2 or T1 based) marker, a diagnosis of AM may be still supported in an appropriate clinical scenario, albeit with less specificity [8]. That is why one non-invasive imaging method may be insufficient to confirm the diagnosis of AM especially when the patient is admitted later than the onset of their symptoms. The recently evolving 2D STE can identify focal or segmental myocardial dysfunction in patients with acute myocarditis, potentially similar to CMR and more accurately than two-dimensional echocardiography, with its global function assessment. The 2D STE findings can be supplemented by the CMR criteria of AM when this procedure is available or the patient’s clinical condition makes it possible to perform it. The aim of the present study was to assess left ventricular myocardial deformation detected by 2D STE in patients with acute myocarditis (AM) in whom cardiac magnetic resonance (CMR) evaluation was performed.

## 2. Methods

### 2.1. Study Population

**Figure 1:**
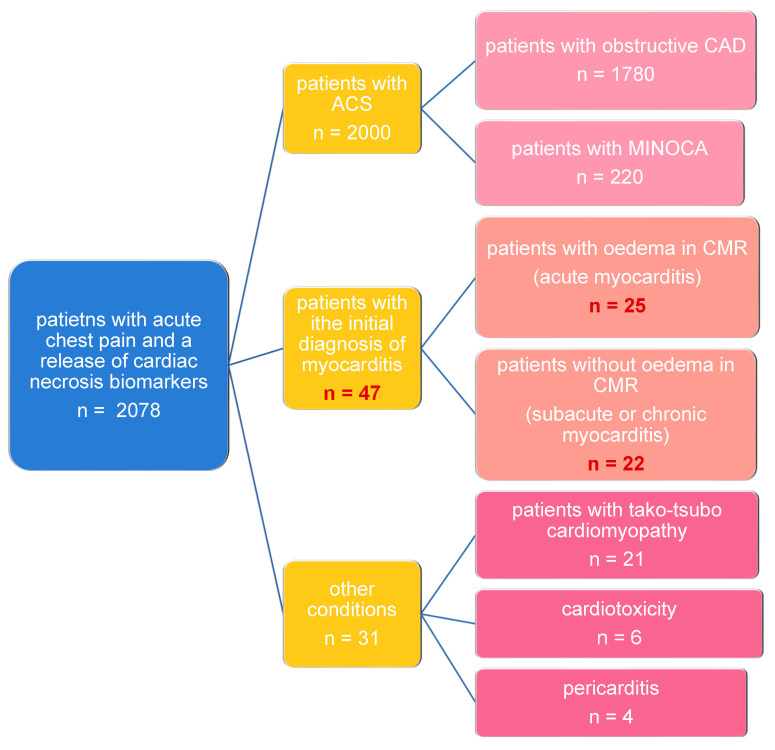
Study flow chart.

A total of 47 consecutive patients with suspicion of AM admitted to the Interventional Cardiology Clinic were prospectively enrolled in our study (Figure 1). The initial diagnosis of AM was established on the basis of a clinical presentation. All the patients presented with severe chest pain starting within 1–2 weeks of a respiratory or gastrointestinal infection, the ST–T wave changes in electrocardiography, and a release of cardiac necrosis biomarkers (hsTnT, CK-MB), mimicking the acute coronary syndrome.

Coronary angiography was performed on all patients to rule out significant coronary artery disease on the first day of hospitalization. Conventional electrocardiography, echocardiography, chest X-ray, and lab tests excluded arrhythmias, congenital valve and myocardial pathologies, and other causes of chest pain. CMR confirmed myocardial inflammation, oedema, and regional necrosis—LGE in 25 patients (53%) meeting the Lake Louise criteria I for the diagnosis of AM [2]. In the remaining patients, only LGE was confirmed in the sub-epicardial or intramuscular localization typical for myocarditis not for ischemia (22 patients, 47%, oedema (−) subgroup). We excluded patients with Fabry disease, hypertrophic cardiomyopathy, arrhythmogenic cardiomyopathy, and cocaine-induced toxic injury from the study as they may have LGE lesions at a location corresponding to myocarditis.

### 2.2. Echocardiography

Echocardiography was performed using a Vivid E9 ultrasound system with a 3.5-Mhz transducer (GE Healthcare, Chicago, IL, USA) on the first day of hospitalization. Images were optimized for gain, compression, depth, and sector width, and acquired at frame rates of 70–90 frames/s. Apical 4- and 2-chamber views, parasternal long-axis views, and parasternal short-axis views at the basal, papillary, and apical ventricular levels were acquired. In each plane, images from three consecutive cardiac cycles were acquired during a breath-hold at end-expiration. The LV end-diastolic and end-systolic volumes were measured from the apical 2- and 4-chamber views, and the ejection fraction (EF) was calculated using the modified Simpson rule.

### 2.3. 2D STE: Two-Dimensional Speckle-Tracking Echocardiography

All participants underwent a comprehensive echocardiographic examination with detailed two-dimensional speckle-tracking echocardiography on the first day of hospitalization. Analysis was performed offline with the aid of a commercially available software package (EchoPAC 201 GE Medical Systems). Speckle-tracking strain analysis is a novel method based on gray-scale two-dimensional (2D) images, which permits the assessment of myocardial deformation in two dimensions. Using apical and parasternal short-axis views, three different patterns of myocardial deformation can be assessed: radial strain (RS) represents the myocardial thickening in a short-axis plane; circumferential strain (CS) represents myocardial shortening in a short-axis plane; and longitudinal strain (LS) represents the myocardial shortening in the long-axis plane. Moreover, circumferential strain was calculated transmurally (transmural GCS, MID GCS) and for myocardial layers—epicardium (epicardial GCS) and endocardium (endocardial GCS), in the assumption that changes in myocarditis are layer-specific with the distribution of oedema in the mid-wall and LGE in the epi- and mid-wall. The ROI (region of interest) was manually adjusted using a point-and-click approach. Images were automatically divided into six standard segments, respectively, and time-strain curves generated from each segment. From the curves, regional and global (by averaging values of all segments) peak and time-to-peak values were obtained [9].

The prevalence of subclinical cardiac systolic dysfunction in our study was defined in relation to previously reported strain values obtained from the Nagata et al. study, defined as GLS < 18.0% to 22%, GCS 18.5% to 23.1%, epicardial GCS 13.3% to 17.3%, endocardial GCS 25.5% to 31.5% in the healthy subjects [10], and GRS 29% to 45.8%, from the Sugimoto et al. study which was also obtained from healthy volunteers [11].

### 2.4. CMR Cardiac Magnetic Resonance

CMR was performed within 48 h of admission on a 1.5 T scanner (Siemens Magnetom Avanto). CMR images were acquired during breath hold and with ECG-gating. Image analysis was performed by an experienced radiologist specialized in the assessment of cardiac images. A combination of 2018 expert recommendation for updated CMR criteria in acute myocardial inflammation (Lake Louise criteria I; 2018 LLC) was evaluated in each case—myocardial oedema (elevated T2-weighted) and non-ischemic injury (elevated T1 and/or ECV and/or LGE) [9]. Regional or global myocardial oedema was identified as an area of high signal intensity with subepicardial or transmural localization in T2-weighted images (T2-weighted STIR (short-tau inversion-recovery) imaging in short-axis orientation—nine slices from the base to the apex of the heart). An intravenous bolus of Gd-DTPA (Gadovist, Bayer Schering Pharma, Berlin, 0.15 mmol per kg body weight) was administered for the purpose of early-enhanced cine imaging as a marker of hyperemia, and late gadolinium enhancement (LGE) was used to identify myocardial fibrosis [2,6,8].

### 2.5. Statistical Analysis

Categorical variables were summarized as frequencies with percentages. The Shapiro–Wilk test was used to assess normally distributed of variables. Due to a lack of normal distribution in the majority of variables, non-parametric tests were used. Continuous variables were expressed as medians with an interquartile range. Correlations were assessed using the Spearman’s rank correlation coefficient. Differences between two continuous variables were compared by using the Mann–Whitney U test. Whereas the chi-squared test with Yates’s correction for continuity was applied to assess differences between categorical variables. Receiver operating characteristics analysis was performed to compare diagnostic performance. All statistical analyses were performed using STATISTICA 13.1 (StatSoft Inc., Tulsa, OK, USA). A *p* value < 0.05 was considered statistically significant.

The study protocol was approved by the local ethics committee (Bioethics Committee at the Medical University of Lodz, Poland) and followed guidelines of the Declaration of Helsinki (RNN/03/20/KE).

## 3. Results

### 3.1. Patient Characteristics

The mean age was thirty-two (IQR 22–43), the prevalence of female gender was 19% (nine patients). All of the patients presented with chest pain. A total of fourteen patients (30%) had a fever on admission, four (9%) had muscle pain typical for a viral infection (myalgia), and twenty-two patients (47%) had symptoms of upper respiratory tract infection (sore throat, cough, rhinitis). C-reactive protein was elevated in 41 patients (87%) and leucocytes (WBC) were elevated in 21 (45%) out of the 47 patients. Furthermore, 33 patients (70%) had a history of infection 7 to 14 days before hospitalization. ST-segment elevation was observed in 36 patients (77%). Cardiac necrosis biomarkers (hsTnT, CK-MB) were elevated in all patients.

The baseline characteristics of the patients are summarized in Table 1. 

### 3.2. CMR Results

CMR was performed in all the patients. EF was slightly but significantly lower in patients with oedema (*p* < 0.028). Additionally, more affected LGE segments were in patients with oedema in comparison with the rest of the patients with statistical significance (*p* < 0.016). CMR parameters are shown in Table 2. 

### 3.3. Echocardiographic Results

**Figure 2:**
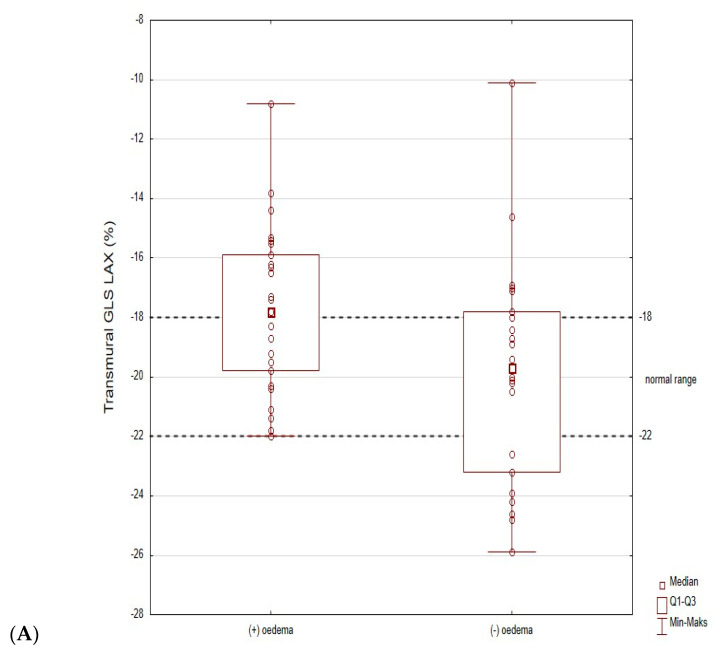

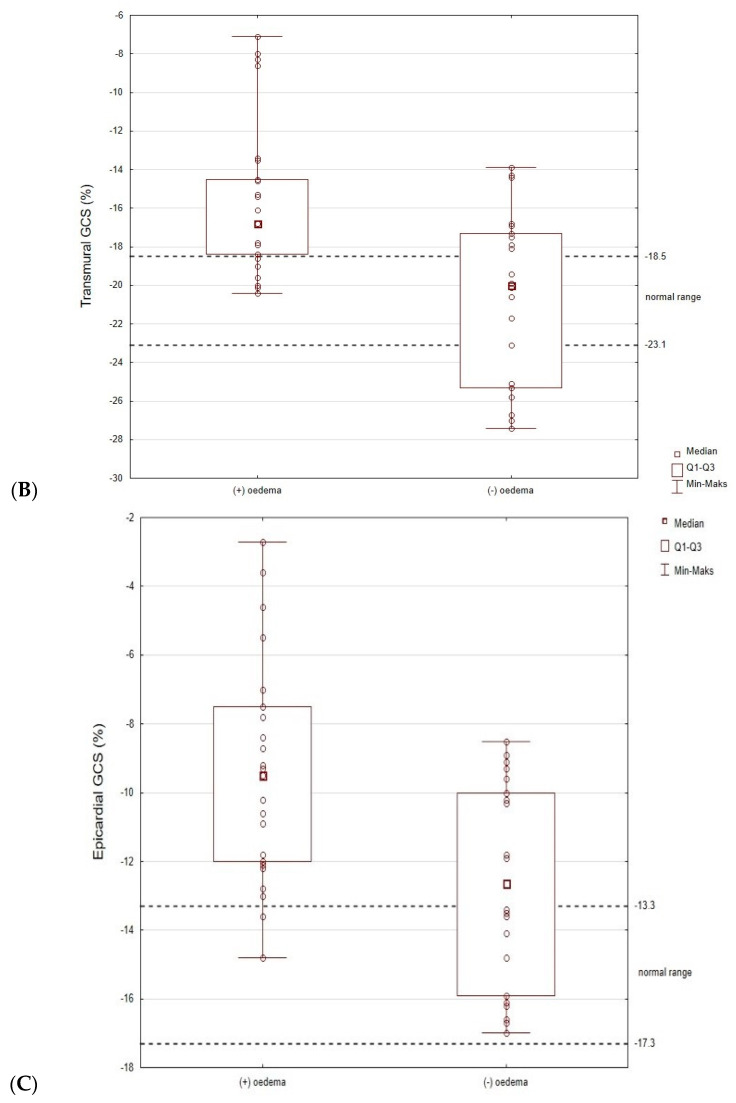

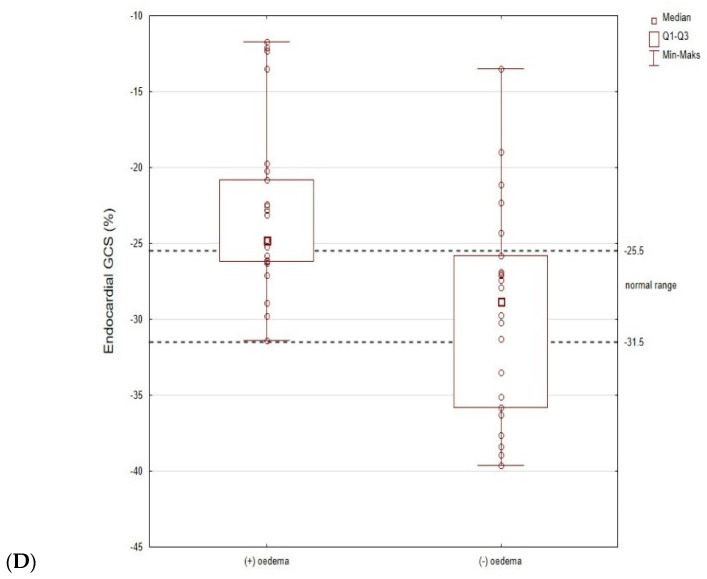
2D STE analysis—(**A**): GLS LAX, (**B**): transmural GCS, (**C**): Epicardial GCS, (**D**): Endocardial GCS broken down into oedema (+) and oedema (−) subgroups (*p* values given indicate differences against subgroups oedema (+) and oedema (−), respectively, GLS LAX 0.041; GCS 0.001, epicardial GCS 0.003, endocardial GCS 0.001). There is normal range between dashed lines.

Echocardiographic parameters with global deformation analysis are shown in Table 3 and Figure 2. 

### 3.4. 2D STE Analysis

In 2D STE analysis, we observed the decreased values of global longitudinal strain (mild only in oedema (+) group), global radial strain (mild only in oedema (+) group), and a more pronounced transmural circumferential strain (−17.9% in the overall population vs. 18.5% as the lower limit of the adopted norm). Noteworthy is small but significant difference in LV EF between subgroups with present or absent oedema in the myocardium measured by classical echocardiography (53 vs. 60%, *p* = 0.009), similar to the magnetic resonance methods (57 vs. 58%, *p* = 0.028). According to the ESC standards, the systolic function of the left ventricle is preserved in those two subgroups (EF above 50%) and clinically, it would be difficult to exploit such a result to differentiate these two groups on the basis of the left ventricular ejection fraction only.

### 3.5. The Subgroup Analysis (Patients with and without Oedema)

In the subgroup analysis, patients with oedema differed significantly in terms of global deformation analysis in the longitudinal strain GLPS LAX (−17.8% vs. −19.7%, *p* < 0.041) and in the circumferential strain (transmural GCS −16.8% vs. −20%, *p* < 0.01) from those without oedema in CMR (Table 2).

**Figure 3:**
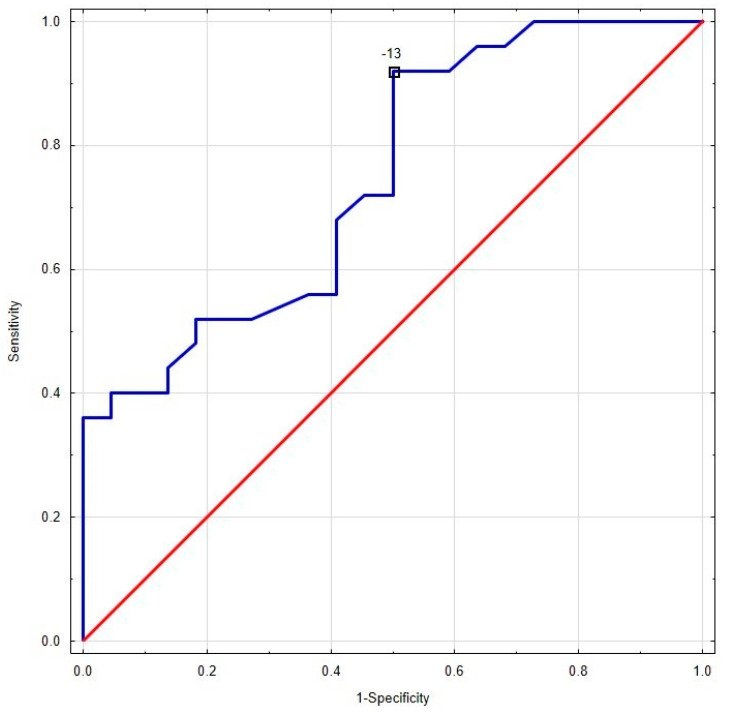
The ROC curve with a cut-off point of 13%, with a sensitivity of 92% and a specificity of 50% for epicardial GCS in patients with myocarditis and oedema confirmed by CMR (AUC 0.747, CI 0.608–0.886, *p* = 0.0005). The blue line—ROC curve for epicardial GCS in oedema (+) AM. The red line—reference line. GCS indicates global circumferential strain; CMR indicates cardiac magnetic resonance; CI indicates confidence interval.

**Figure 4:**
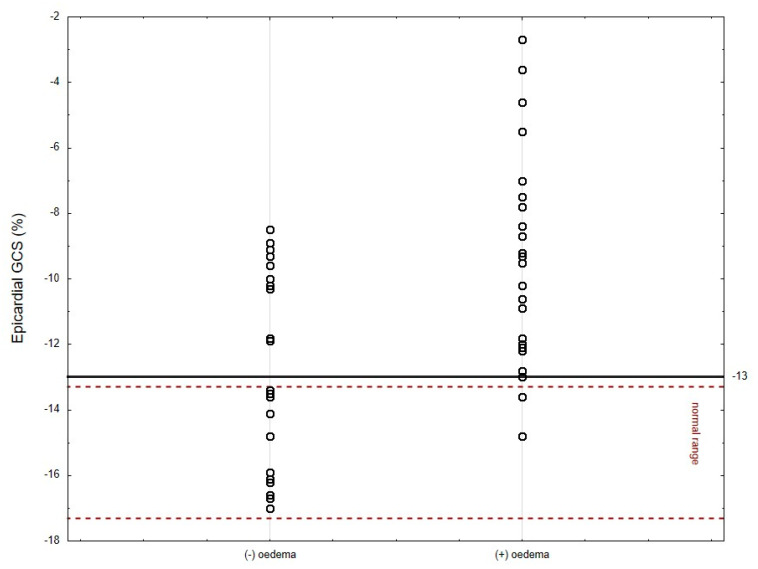
Epicardial GCS with a cut-off point of 13.0% dividing patients into two groups—with or without oedema confirmed by CMR (*p* = 0.003). The caption of the figure explains the solid line. There is a normal range between dashed lines. GCS indicates global circumferential strain; CMR indicates cardiac magnetic resonance.

**Figure 5:**
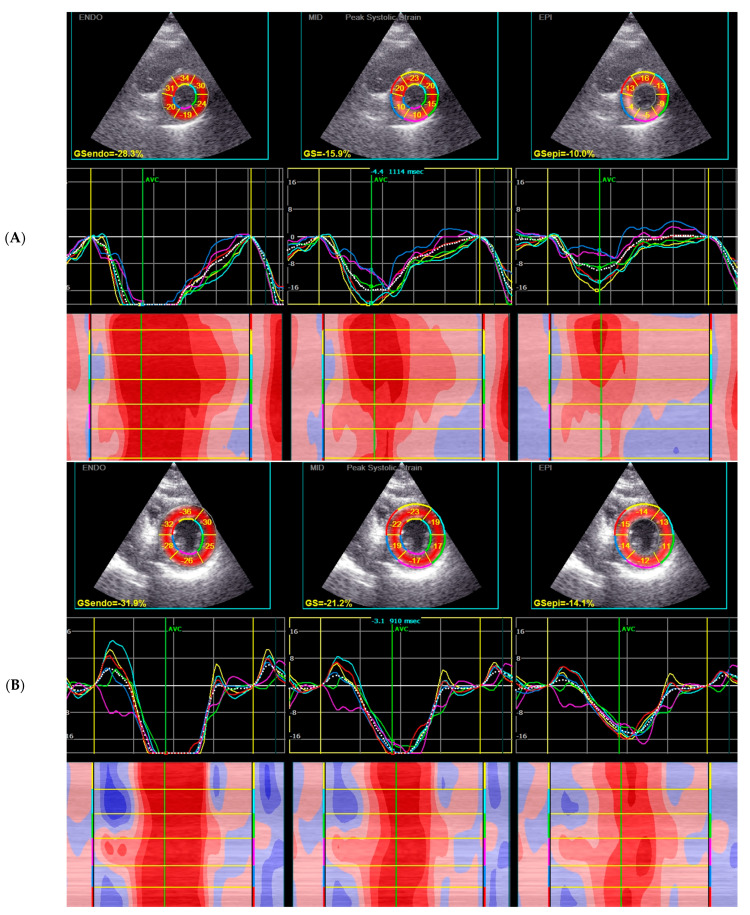
2D STE findings—GCS (epi and endo-layers and MID—transmural GCS) in a representative patient with oedema (**A**) and without oedema (**B**) confirmed in CMR.

**Figure 6:**
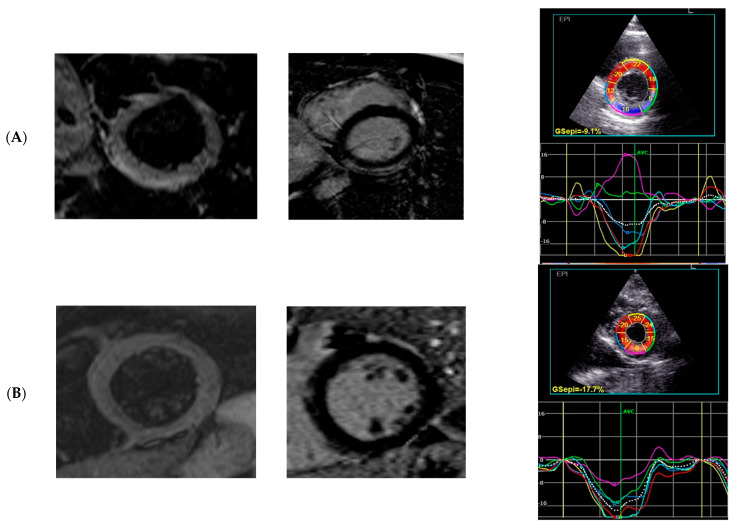
A representative patient with oedema (**A**) and without oedema (**B**); basal segments in short axis: CMR—oedema in T2-weighted image, LGE positive in T1 image, 2D STE GCS Epi, respectively.

As shown in Figure 2, the results of most patients in the oedema (−) group (Q1–Q3) are within the adopted norms. The distribution of the results is different for the oedema (+) group, where the transmural GCS results are below the norm (the results for the Q1 are borderline normal). Additionally, for endocardial GCS, the Q1 results overlap with accepted norms. Only the epicardial GCS Q1–Q3 results are well below adopted norms. Moreover, the median value for epicardial GCS differs significantly between these two populations (−9.5% for oedema (+) group vs. −12.7% for oedema (−) group, *p* = 0.003, Figure 2, Figure 3 and Figure 4). Based on the ROC curves, the epicardial GCS was found to be the diagnostic factor for oedema with a cut-off point of −13.0%, with a sensitivity of 92% and a specificity of 50% (AUC 0.747, CI 0.608–0.886, *p* = 0.0005) (Figure 5). Twenty-three patients with epicardial GCS 13.0% or less had oedema confirmed by CMR (Figure 6).

The area between the dashed lines: normal range for GLS LAX, 18 to 22%; for GCS, 18.5 to 23.1%; for epicardial GCS, 13.3 to 17.3%; for endocardial GCS, 25.5 to 31.5%.

2D STE indicates two-dimensional speckle-tracking echocardiography; GLS LAX indicates global longitudinal strain in long axis; GCS indicates global circumferential strain.

2D STE indicates two-dimensional speckle-tracking echocardiography; GCS indicates global circumferential strain; CMR indicates cardiac magnetic resonance.

CMR indicates cardiac magnetic resonance; LGE indicates late gadolinium enhancement; 2D STE indicates two-dimensional speckle-tracking echocardiography; GCS Epi indicates global circumferential strain epicardial layer.

## 4. Discussion

The diagnosis of AM can be challenging because of its clinical presentation mimicking other cardiologic diseases and an inaccessibility of the main non-invasive diagnostic imaging modality—CMR—in the early phase of the disease in several institutions. Some patients have contraindications to CMR, e.g., implanted metal elements in the body or a history of allergy to contrast agents. EMB, still the gold-standard method for the diagnosis of AM, is reserved for patients with hemodynamic instability, presenting as acute HF or cardiogenic shock, and rapidly deteriorating [12].

So far, little is known about the transition time from acute to subacute and chronic disease in myocarditis. Patients report at different times from the onset of infectious symptoms, at different stages of the disease, and usually the infectious agent is unknown. The oedema in AM can be best visualized during the first days of the disease. In the study by Monney et al., myocardial oedema decreased from 84% to 39% after a >3-week follow-up [13]. In the study by Luetkens et al., myocardial oedema decreased also from 88% to 35% at 4-to-8-week follow-up and the semiquantitative T2 ratio—the second surrogate for the presence of myocardial oedema—normalized within 3 weeks [14]. The authors found a convergence between the duration of oedema and the pathophysiological course of viral myocarditis, in which cardiac damage is mainly described during the first week (the acute phase of the disease) and lasts 1 to 4 weeks (the subacute phase of the disease) [14,15]. Due to the resolution of oedema over time, new indicators of acute, subacute, or persistent myocarditis are sought. In the Luetkens et al. study, myocardial T1 and T2 relaxation times, not included in the original Lake Louise criteria, were the only parameters of active inflammation/oedema that could discriminate between acute, convalescent myocarditis, and healthy controls. The authors emphasized that in contrast to T2-weighted imaging, both parameters were significantly elevated compared to controls after a 4-to-8-week follow-up, making them more sensitive in detecting myocardial oedema [14].

The diagnostic value of echocardiography still has been limited by the fact that many patients with less severe AM have a normal echocardiogram. Novel strain parameters (2D STE) allow it to compete with CMR due to setting the diagnosis on the early stages of the disease with better availability and the possibility of performing a bedside examination. Strain analysis by 2D STE appears to be very informative in AM. It facilitates diagnosis and may be a new tool to detect and monitor inflammatory myocardial injuries (oedema, myocardial fibrosis) during the course of AM (Figure 3 and Figure 4). The left ventricle myocardium has a complex architecture. Changes in the myocarditis are layer-specific with the distribution of oedema in the mid-wall and LGE in the epi- and mid-wall (circumferential fibers predominate, mostly affected GCS), in contrast with acute coronary syndromes where the layer mainly affected by ischemia is the sub-endocardium, where longitudinal fibers predominate (only mild changes in GLS values in our oedema (+) group). This agrees with previous findings describing the site of injury in peri-myocarditis vs. ischemic injuries confirming the initial diagnosis of myocarditis. We can presume that a more affected GLS in a situation when the coronary arteries seem normal may suggest MINOCA rather than myocarditis. This approach requires further research.

Considering regional changes in strain values, we can expect that the segmental changes may correspond to the location of the oedema. Often, these changes are patchy and go beyond the vascularization of a particular coronary artery, in contrast to the ischemic lesions with their predictable vascularization pattern.

Until now, most of the studies on myocarditis and STE assessment have analyzed the relationship between changes in the myocardium found in CMR (oedema, LGE) and only with GLS [16,17,18,19,20]. In line with these studies, we also observed the decreased values of global longitudinal strain (mild only in oedema (+) group). However, in our research we also observed the decreased values of global radial strain (mild only in oedema (+) group) and a more pronounced transmural circumferential strain. The results of STE analysis of most of the patients in the oedema (−) group were within the adopted norms from previous studies [12,13]. The distribution of results was different for the oedema (+) group, where transmural GCS results were below the norm (the results for the Q1 were borderline normal), and for endocardial GCS, the Q1 results overlapped with accepted norms. Only the epicardial GCS Q1–Q3 results were well below adopted norms (Figure 1). Moreover, based on the ROC curves, the epicardial GCS was discovered to be the best diagnostic factor for oedema with a cut-off point of 13.0%, with a sensitivity of 92% and a specificity of 50% (AUC 0.747, *p* = 0.0005). A total of 23 out of 25 (92%) patients with an acute phase of myocarditis and an epicardial GCS of 13.0% or less had oedema confirmed by CMR.

In contrast, in the Logstrup et al. study [20], 2D STE showed high correlations between GLS and the amount and localization of the oedema found in CMR, but not between GCS and oedema in patients with AM. However, Logstrup’s research has not analyzed the CS by a multi-layer model of heart muscle (epi-, endo-, and transmural CS) as in our study. Similar to our research, the segmental strain decrease was predominantly localized in the infero-postero-lateral segments of the LV, where the oedema was mainly localized on CMR [20].

Moreover, in our study, patients with the presence of myocardial oedema compared to patients without it had higher hsTnT levels (*p* = 0.023) and CK-MB maximal values (*p* = 0.049). Conversely, there was no such relationship between C-reactive protein and the presence of oedema in CMR (*p* = 0.346). These relationships require further studies because they have not been analyzed in the available literature so far.

The study of Leitman et al. [17] revealed a high correlation between the regional strain values and the presence of delayed enhancement in the same LV segments in patients with AM or perimyocarditis. Similar findings were described by Kostakou et al. [21]. In their study, regional LV systolic dysfunction assessed by STE corresponded to the LGE changes in the same LV segments. The authors hypothesized that STE could be a promising method for the detection of LV regional fibrosis. Sperlongano et al. [18] confirmed these results. In their study, segmental peak systolic longitudinal strain was significantly different between segments with and without LGE on CMR and a segmental strain of 12% identified a scar with a sensitivity of 79% and a specificity of 84%. Similar findings emerged from the study of Di Bella, where the longitudinal strain was decreased in segments with positive LGE, while the circumferential and radial strain did not change significantly [22]. All our patients had LGE found in CMR; however, our study was devoted to global strain and layer-specific CS values but not segmental strain assessment.

It is emphasized that one non-invasive imaging method can be insufficient to confirm the diagnosis of AM [20]. In that scenario, 2D STE may be the first method to detect subtle but clinically relevant LV dysfunction even in less-severe AM and can be supplemented by the CMR findings when this procedure is available or the patient’s clinical condition makes it possible to perform it. Recent studies revealed good inter-technique agreement in strain measurements between 2D STE and CMR techniques [17,21,23] and a correlation between novel echocardiography parameters and oedema in CMR [24], similar to our study. The above-mentioned studies on the identification and localization of a scar after myocarditis (segments with possible LGE) with the usage of segmental strain values also are promising in terms of monitoring the course and recovery from myocarditis. It may be clinically relevant to perform subsequent non-invasive 2D STE measurements in the course of myocarditis instead of the CMR investigations which involve loading the patient with contrast agents. This approach requires further research.

Moreover STE, especially epicardial GCS, can serve as a good indicator for selecting a site for specimen collection for histopathological analysis in patients for whom EMB is indicated due to a fulminant or progressive course of the disease.

## 5. Conclusions

This study has shown that 2D STE can help to set the diagnosis of AM in patients with acute chest pain with normal coronary angiography. The epicardial GCS can serve as a diagnostic factor for oedema, as 23 out of 25 (92%) patients with epicardial GCS of 13.0% or less, with a sensitivity of 92% and a specificity of 50%, had oedema confirmed by CMR. In patients presenting with signs of acute myocarditis (oedema in CMR), the epicardial GCS is modified in comparison with a subgroup without oedema; therefore, this parameter could be used to improve the performance of ultrasound. Furthermore, multi-layer 2D STE analysis may be a promising method for monitoring the course and resolution of myocarditis instead of repeated CMR investigations.

## 6. Limitations

The present study has several limitations. Our study represents a single-center experience in a relatively small sample size. We did not confirm the diagnosis by performing EMB, which is the gold standard for the diagnosis of AM. The main findings of the present study are valid in the subgroup of patients who presented with the specific infarct-related symptoms. In addition, the CMRs were performed 2 days after the initial 2D STE measurements. We had no opportunity to calculate T1 and T2 relaxation times in patients with positive LGE but without oedema in CMR. Furthermore, the LGE lesions in patients without oedema in CMR could correspond to old lesions of myocardial injury (it could be a subacute or chronic phase of myocarditis or even a past myocarditis). This may be a confounding factor in the study.

Since 2D STE strain values may differ between several vendors, it would be useful to confirm the findings of our study through a different software.

## Figures and Tables

**Table 1:** Baseline characteristics of patients.

Parameters	All Patients Me (IQR)	Oedema (+) Me (IQR)	Oedema (−) Me (IQR)	*p*
Age, years	32 (22–43)	29 (21–41)	32.5 (24–45)	0.321
Systolic blood pressure, mmHg	129 (119–138)	130 (120–135)	124 (115–140)	0.601
Diastolic blood pressure, mmHg	75 (70–85)	77 (70–84)	71 (70–85)	0.424
Heart rate, bpm	80 (70–90)	82 (70–90)	75 (64–90)	0.316
Peak C-reactive protein, mg/L	42.4 (12–102.7)	43.3 (25.3–112)	37.8 (11–101.2)	0.348
WBC, 10^6^/L	9.5 (7.91–12.06)	9.5 (8.1–11.9)	9.3 (7.2–12.4)	0.725
Hgb, g/dL	14.5 (13.8–15.4)	14.6 (14.1–15.4)	14.5 (13.4–15.2)	0.741
HCT, %	42.6 (40.1–45.6)	43 (41–45.6)	41.9 (38.6–45.3)	0.301
Peak TnT hs, ng/L	639.5 (118–1252)	741 (376–1315)	322 (10–949)	0.025
Peak CK-MB max, ng/L	23.59 (4.2–102.7)	26.3 (15.1–52.5)	11.7 (1.3–38.7)	0.049
Plasma glucose, mmol/L	6.17 (5.51– 6.96)	5.9 (5.51–6.96)	6.2 (5.66–6.82)	0.877
eGFR, mL/min/1.73 m^2^	112.1 (96.1–118.3)	112.8 (104.6–117.9)	104.5 (94.9–118.3)	0.326

WBC (White Blood Count); Hgb (Hemoglobin); HCT (Hematocrit); TnT (Troponin T); CK-MB (Creatine Kinase MB isoform); eGFR (estimated Glomerular Filtration Rate).

**Table 2:** Cardiac magnetic resonance parameters.

Parameters	All Patients Me (IQR)	Oedema (+) Me (IQR)	Oedema (−) Me (IQR)	*p*
EF, %	58.0 (52–60)	57.0 (51–60)	58 (57–62)	0.028
LV segments with oedema, n	-	2 (1–2)	0 (−)	0.000
LV segments with LGE, n		4 (4–6)	4 (2–4)	0.016
Pericardial effusions/pericardial abnormalities, n	0	0	0	-

LV (Left Ventricle); LGE (Late Gadolinium Enhancement).

**Table 3:** Echocardiographic parameters.

Parameters	All Patients Me (IQR)	Oedema (+) Me (IQR)	Oedema (−) Me (IQR)	*p*
EF, %	58 (52–60)	53.0 (48–60)	60 (57–61)	0.009
LVIDD, mm	50 (46–53)	51 (47–53)	49 (45–51)	0.082
LVIDS, mm	34 (30–36)	35 (30–37)	32 (29–35)	0.314
LV EDV, mL	110 (105–118)	114 (107–123)	107.5 (101–115)	0.038
LV ESV, mL	55 (53–58)	55 (53–59)	55 (53–58)	0.759
PWd, mm	10 (9–11)	10 (9–11)	9.5 (9–10)	0.907
PWs, mm	14 (13–15)	14 (13–15)	14 (13–15)	0.649
IVSd, mm	10 (10–11)	10 (9–11)	10 (10–12)	0.680
IVSs, mm	14 (13–15)	14 (13–15)	14,5 (13–15)	0.519
LAVI, mL/m^2^	30 (27–35)	29 (27–40)	31 (27–35)	0.872
E/A ratio	1.4 (1.2–1.7)	1.5 (1.2–1.7)	1.4 (1.2–1.5)	0.563
E/E’ ratio	7 (6–8)	7 (5–7)	7 (7–9)	0.058
TAPSE, mm	23 (21–26)	23 (20–25)	23,5 (22–26)	0.191
Global deformation analysis				
GLPS Avg, %	−19.1 (−20.4–−16.2)	−17.7 (−20.1–−16.0)	−19.5 (−21.51–−18.5)	0.074
GLPS LAX, %	−18.7 (−20.5–−16.5)	−17.8 (−19.8–−15.9)	−19.7 (−23.2–−17.8)	0.041
GLPS A2C, %	−19.85 (−21.2–−16.9)	−18.35 (−21.0–−16.6)	−20.3 (−21.4–−18.2)	0.25
GLPS A4C, %	−18.3 (−20–−15.7)	−17.0 (−19.4–−15.6)	−19.4 (−20.1–−17.7)	0.055
GRS, %	29.2 (24.6–38.8)	28.6 (24.3–36.5)	33.5 (26.1–40.6)	0.17
GCS, %	−17.9 (−20.1–−15.3)	−16.8 (−18.4–−14.5)	−20.0 (−25.3–−17.3)	0.001
GCS MID systolic strain	−17 (−14.4–−19.4)	−15.7 (−17.2–−13.7)	−19.6 (−23.8–−16.6)	0.000
GCS EPI systolic strain	−10.6 (−13.5–−8.9)	−9.5 (−12.0–−7.5)	−12.7 (−15.9–−10.0)	0.003
GCS ENDO systolic strain	−26.2 (−29.8–−22.4)	−24.8 (−26.2–−20.8)	−28.8 (−35.8–−25.8)	0.001

EF (Ejection fraction); LVIDD (Left Ventricular Internal Diameter – Diastole); LVIDS (Left Ventricular Internal Diameter – Systole); LV EDV (Left Ventricular End-Diastolic Volume); LV ESV (Left Ventricular End-Systolic Volume); PWd (Posterior Wall diastole); PWs (Posterior Wall systole); IVSd (Intraventricular Septum diastole); IVSs (Intraventricular Septum systole); LAVI (Left Atrium Volume Index); E/A ratio (ratio of early and late diastolic inflow velocities); E/E’ ratio (ratio of early diastolic inflow velocity and early diastolic movement of the mitral annulus); TAPSE (Tricuspid Annular Plane Systolic Excursion); GLPS Avg (Global Longitudinal Peak Strain Average); GLPS LAX (Global Longitudinal Peak Strain Long-Axis); GLPS A2C (Global Longitudinal Peak Strain Two-Chamber-Axis); GLPS A4C (Global Longitudinal Peak Strain Four-Chamber-Axis); GRS (Global Radial Strain); GCS (Global Circumferential Strain); GCS MID systolic strain (Global Circumferential Strain mid-myocardial systolic strain); GCS EPI systolic strain (Global Circumferential Strain epicardial systolic strain); GCS ENDO systolic strain (Global Circumferential Strain endocardial systolic strain).

## Data Availability

The data presented in this study are available on request from the corresponding author.
